# Supplementary material for: Rapid assembly of SARS-CoV-2 genomes reveals attenuation of the Omicron BA.1 variant through NSP6
Source: Nat Commun. 2023 Apr 21;14:2308. doi: 10.1038/s41467-023-37787-0 (PMC10120482; doi:10.1038/s41467-023-37787-0)
Supplement: Supplementary file 1 — Supplementary Information file [file 41467_2023_37787_MOESM1_ESM.pdf]

Supplementary Information

Rapid assembly of SARS-CoV-2 genomes reveals attenuation of the Omicron BA.1 variant through NSP6

Taha Y. Taha[1,#,*], Irene P. Chen[1,2,#], Jennifer M. Hayashi[1,#], Takako Tabata[1,#], Keith Walcott[1], Gabriella R. Kimmerly[1], Abdullah M. Syed[1,3], Alison Ciling[1,3], Rahul K. Suryawanshi[1], Hannah S. Martin[1,6], Bryan H. Bach[3], Chia-Lin Tsou[1], Mauricio Montano[1], Mir M. Khalid[1], Bharath K. Sreekumar[1], G. Renuka Kumar[1], Stacia Wyman[3], Jennifer A. Doudna[1,3-8], and Melanie Ott[1,2,9,*]

[#]These authors contributed equally to this work
[1]Gladstone Institutes, San Francisco, CA, USA
[2]Department of Medicine, University of California, San Francisco, CA, USA
[3]Innovative Genomics Institute, University of California, Berkeley, Berkeley, CA, USA
[4]Department of Molecular and Cell Biology, University of California, Berkeley, CA, USA
[5]Howard Hughes Medical Institute, University of California, Berkeley, Berkeley, CA, USA
[6]Department of Chemistry, University of California, Berkeley, Berkeley, CA, USA
[7]Molecular Biophysics and Integrated Bioimaging Division, Lawrence Berkeley National Laboratory, Berkeley, CA, USA
[8]California Institute for Quantitative Biosciences (QB3), University of California, Berkeley, Berkeley, CA, USA
[9]Chan Zuckerberg Biohub – San Francisco, San Francisco, CA, USA
*Correspondence to: Taha Y. Taha, taha.taha@gladstone.ucsf.edu; Melanie Ott, melanie.ott@gladstone.ucsf.edu

A

	Overhang				
	5′	3′	nt	nt	ORF
**F1**	ATTA	GTGC	1	2721	ORF1a (nsp1&2)
**F2**	GTGC	GAGA	2718	5454	ORF1a (nsp3)
**F3**	GAGA	GTAA	5451	8556	ORF1a (nsp3)
**F4**	GTAA	TCTA	8553	11846	ORF1a (nsp4–6)
**F5**	TCTA	TGCA	11843	15090	ORF1a (nsp7-11), ORF1ab (nsp12)
**F6**	TGCA	GCTG	15087	18043	ORF1ab (nsp12&13)
**F7**	GCTG	CAAT	18040	21564	ORF1ab (nsp14–16)
**F8**	CAAT	GAAC	21561	25390	S
**F9**	GAAC	ACGA	25387	27891	ORF3a/b, E, M, ORF6, ORF7a/b
**F10**	ACGA	AAAA	27888	29908	ORF8, N, ORF9b/c, ORF10

B

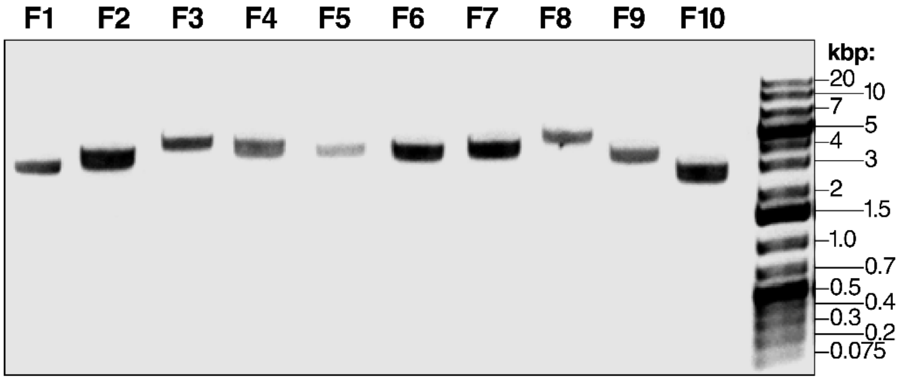

Supplementary Figure 1. SARS-CoV-2 fragment design and quality.

(A) Coordinates of the 10 SARS-CoV-2 fragments and the sequences of the 5′ and 3′ overhangs when the fragments were digested by BsaI. Coordinates are based on the WA1 sequence.

(B) Agarose gel electrophoresis of the 10 fragments from A after PCR amplification and clean up. 200 ng of each fragment was loaded on the gel. The gel is representative of 3 independent fragment amplification reactions.

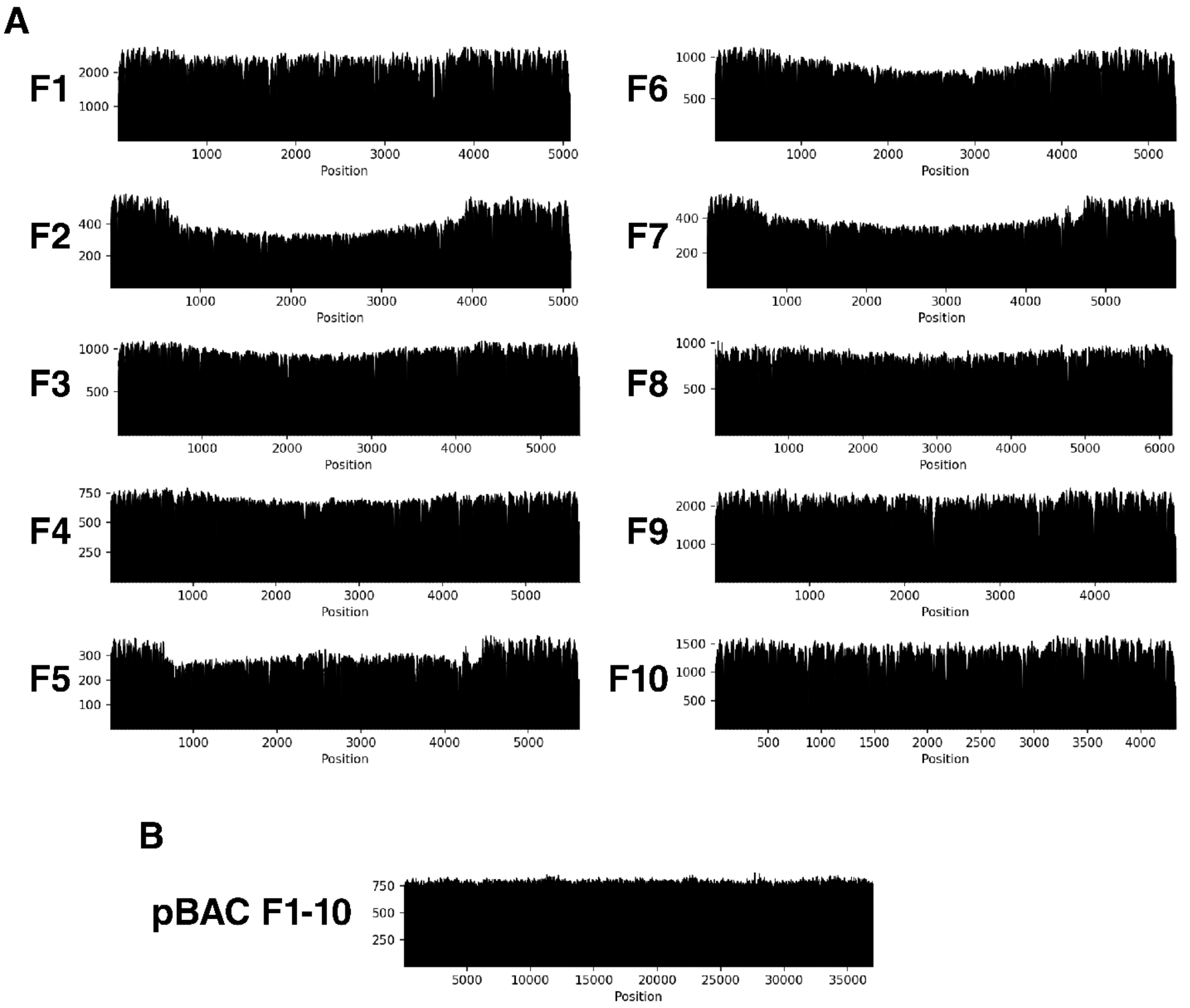

Supplementary Figure 2. Nanopore sequencing of the 10 SARS-CoV-2 genome fragment plasmids and the pBAC SARS-CoV-2 plasmid.

(A) Representative coverage (# reads mapped per base) plots for each of the 10 SARS-CoV-2 genome fragment plasmids. Sequencing was done through Primordium Labs whole plasmid sequencing service. Plots were generated as part of the sequencing service.

(B) Representative coverage (# reads mapped per base) plot of the pBAC SARS-CoV-2 plasmid. Sequencing was done through Primordium Labs whole “Large” plasmid sequencing service. Plot was generated as part of the sequencing service.

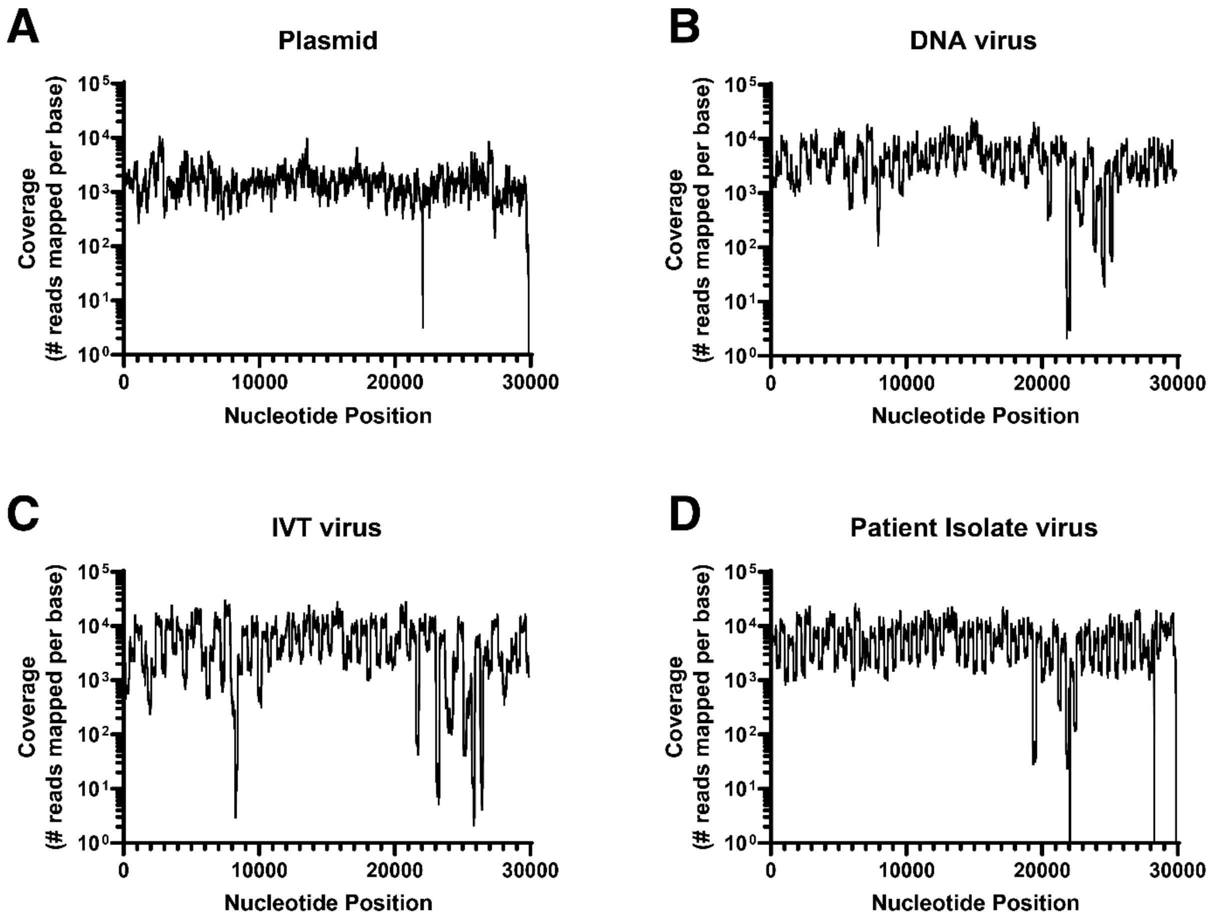

Supplementary Figure 3. NGS sequence verification of pBAC SARS-CoV-2 plasmid and patient isolate, DNA- and RNA-launched viruses.

Representative coverage (# reads mapped per base) plots of the plasmid used to generate the viruses in Fig. 2 (A), including DNA- (B) and RNA-launched (C) viruses and the patient isolate virus (D). All sequencing was done using the ARTIC Network's protocol.

Source data are provided as a Source Data file.

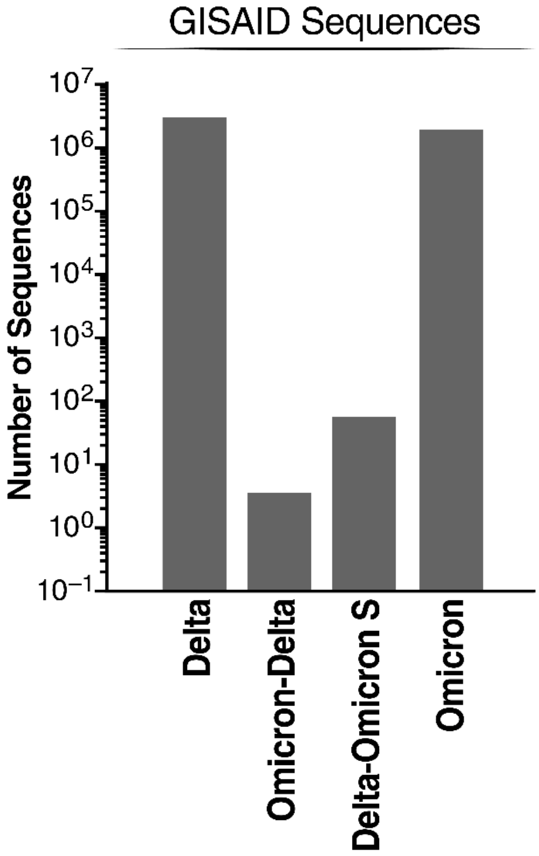

Supplementary Figure 4. Abundance of SARS-CoV2 Delta, Omicron, and Delta-Omicron recombinant sequences.

Sequences were extracted from the GISAID database using key mutations specific to each of the Delta and Omicron variants (see Fig. 3 for more details). Omicron-Delta indicates an ORF1ab and ORF2-10 recombinant.

Source data are provided as a Source Data file.

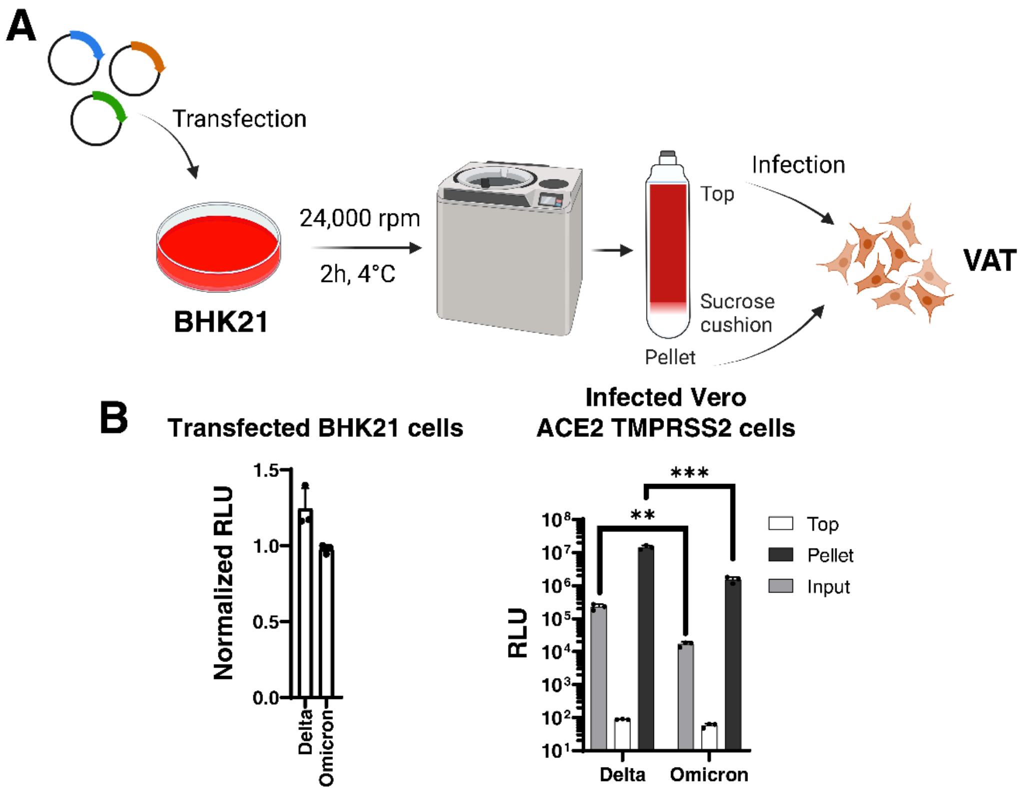

Supplementary Figure 5. Concentration and validation of SARS-CoV-2 replicon-containing particles.

(A) Workflow for the concentration and validation of SARS-CoV-2 replicon-containing particles. VAT: Vero cells stably expression ACE2 and TMPRSS2. The image was created with Biorender.

(B) Luciferase readout from transfected BHK21 and infected Vero ACE2 TMPRSS2 cells of Delta and Omicron replicons. Data shown are average ± SD of three independent transfections and concentration experiments, and pairwise comparisons between the Delta and Omicron variant were done with two-sided Student's T-test. **, $p<0.01$; ***, $p<0.001$

Source data are provided as a Source Data file.

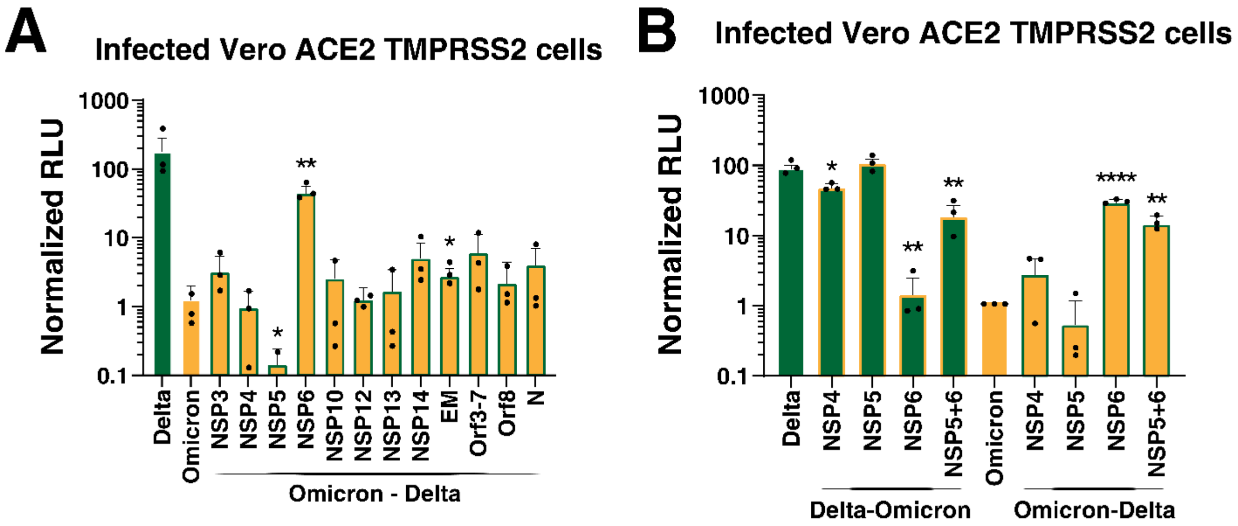

Supplementary Figure 6. Omicron mutations in NSP6 reduce viral RNA replication.

(A) Luciferase readout from infected Vero ACE2 TMPRSS2 cells with supernatant from BHK21 cells transfected with Delta, Omicron, and Omicron-Delta recombinants replicons as indicated and an Omicron spike and nucleocapsid expression vectors. Average of three independent experiments analyzed in duplicate ± SD are shown, and pairwise comparisons were made relative to the Omicron variant by two-sided Student's T-test.

(B) Luciferase readout from infected Vero ACE2 TMPRSS2 cells with supernatant from BHK21 cells transfected with Delta, Delta-Omicron recombinants, Omicron, and Omicron-Delta recombinants replicons as indicated and an Omicron spike and nucleocapsid expression vectors. Average of three independent experiments analyzed in duplicate ± SD are shown, and pairwise comparisons were made relative to the Omicron variant by two-sided Student's T-test.

*, $p<0.05$; **, $p<0.01$; ****, $p<0.0001$ by two-sided Student's T-test.

Source data are provided as a Source Data file.
