# Supplementary material for: Rapid assembly of SARS-CoV-2 genomes reveals attenuation of the Omicron BA.1 variant through NSP6
Source: Nat Commun. 2023 Apr 21;14:2308. doi: 10.1038/s41467-023-37787-0 (PMC10120482; doi:10.1038/s41467-023-37787-0)
Supplement: Supplementary file 2 — Reporting Summary [file 41467_2023_37787_MOESM2_ESM.pdf]

nature portfolio

Corresponding author(s): Melanie Ott, Taha Y. Taha

Last updated by author(s): Mar 24, 2023

# Reporting Summary

Nature Portfolio wishes to improve the reproducibility of the work that we publish. This form provides structure for consistency and transparency in reporting. For further information on Nature Portfolio policies, see our Editorial Policies and the Editorial Policy Checklist.

## Statistics

For all statistical analyses, confirm that the following items are present in the figure legend, table legend, main text, or Methods section.

| n/a | Confirmed | |
|---|---|---|
| ☐ | ☒ | The exact sample size (*n*) for each experimental group/condition, given as a discrete number and unit of measurement |
| ☐ | ☒ | A statement on whether measurements were taken from distinct samples or whether the same sample was measured repeatedly |
| ☐ | ☒ | The statistical test(s) used AND whether they are one- or two-sided<br>*Only common tests should be described solely by name; describe more complex techniques in the Methods section.* |
| ☒ | ☐ | A description of all covariates tested |
| ☒ | ☐ | A description of any assumptions or corrections, such as tests of normality and adjustment for multiple comparisons |
| ☐ | ☒ | A full description of the statistical parameters including central tendency (e.g. means) or other basic estimates (e.g. regression coefficient) AND variation (e.g. standard deviation) or associated estimates of uncertainty (e.g. confidence intervals) |
| ☐ | ☒ | For null hypothesis testing, the test statistic (e.g. *F*, *t*, *r*) with confidence intervals, effect sizes, degrees of freedom and *P* value noted<br>*Give P values as exact values whenever suitable.* |
| ☒ | ☐ | For Bayesian analysis, information on the choice of priors and Markov chain Monte Carlo settings |
| ☒ | ☐ | For hierarchical and complex designs, identification of the appropriate level for tests and full reporting of outcomes |
| ☒ | ☐ | Estimates of effect sizes (e.g. Cohen's *d*, Pearson's *r*), indicating how they were calculated |

*Our web collection on statistics for biologists contains articles on many of the points above.*

## Software and code

Policy information about availability of computer code

| | |
|---|---|
| Data collection | No software was used for data collection. |
| Data analysis | GraphPad Prism version 9.4.1 for Windows, GraphPad Software, San Diego, California USA, www.graphpad.com<br>Geneious Prime version 2022.2.1. https://www.geneious.com |

For manuscripts utilizing custom algorithms or software that are central to the research but not yet described in published literature, software must be made available to editors and reviewers. We strongly encourage code deposition in a community repository (e.g. GitHub). See the Nature Portfolio guidelines for submitting code & software for further information.

## Data

Policy information about availability of data

All manuscripts must include a data availability statement. This statement should provide the following information, where applicable:

- Accession codes, unique identifiers, or web links for publicly available datasets
- A description of any restrictions on data availability
- For clinical datasets or third party data, please ensure that the statement adheres to our policy

All data supporting the findings of the present study are available in the article, extended data and supplementary figures, or are available from the corresponding author on request. Source data are provided with this paper.

## Human research participants

Policy information about studies involving human research participants and Sex and Gender in Research.

| | |
|---|---|
| Reporting on sex and gender | No information on sex or gender have been collected in this study. |
| Population characteristics | Not applicable |
| Recruitment | Not applicable |
| Ethics oversight | Not applicable |

Note that full information on the approval of the study protocol must also be provided in the manuscript.

# Field-specific reporting

Please select the one below that is the best fit for your research. If you are not sure, read the appropriate sections before making your selection.

[x] Life sciences [ ] Behavioural & social sciences [ ] Ecological, evolutionary & environmental sciences

For a reference copy of the document with all sections, see nature.com/documents/nr-reporting-summary-flat.pdf

# Life sciences study design

All studies must disclose on these points even when the disclosure is negative.

| | |
|---|---|
| Sample size | No sample size calculation was performed. For experiments involving mice, three mice were selected for each group to assess if there are biologically significant differences in the DNA- and RNA-launched viruses compared with patient isolate virus. |
| Data exclusions | No data exclusions were performed. |
| Replication | At least three independent biological replicate experiments were used to ensure reproducibility of the results, and replicate experiments successfully reproduced the findings in all experiments. The exact number of independent experiments for each figure has been defined in the figure legend. |
| Randomization | This is not relevant to this study because no human subjects were involved and all the mice or cells utilized in the study were isogenic and therefore no randomization was necessary. |
| Blinding | Blinding was not done for this study as it was difficult given the stark difference in replicative capacity of Delta and Omicron variants. |

# Reporting for specific materials, systems and methods

We require information from authors about some types of materials, experimental systems and methods used in many studies. Here, indicate whether each material, system or method listed is relevant to your study. If you are not sure if a list item applies to your research, read the appropriate section before selecting a response.

### Materials & experimental systems

| n/a | Involved in the study | |
|---|---|---|
| [ ] | [x] | Antibodies |
| [ ] | [x] | Eukaryotic cell lines |
| [x] | [ ] | Palaeontology and archaeology |
| [ ] | [x] | Animals and other organisms |
| [x] | [ ] | Clinical data |
| [ ] | [x] | Dual use research of concern |

### Methods

| n/a | Involved in the study | |
|---|---|---|
| [x] | [ ] | ChIP-seq |
| [x] | [ ] | Flow cytometry |
| [x] | [ ] | MRI-based neuroimaging |

## Antibodies

| | |
|---|---|
| Antibodies used | Anti-FLAG: Sigma Aldrich F1804, Mouse, M2 clone<br>Anti-dsRNA: Cell Signaling 76651, Mouse, J2 clone<br>Anti-Mouse AlexaFluor 488: ThermoFisher A-21202, Donkey, polyclonal<br>All dilutions are indicated in the Methods section of the manuscript. |

| | |
|---|---|
| Validation | - Anti-FLAG: Antibody was validated for IF by manufacturer using untransfected controls. See data at https://www.sigmaaldrich.com/deepweb/assets/sigmaaldrich/product/documents/144/194/vol6_iss2_antiflag_m2.pdf<br>In addition, antibody was validated by including untransfected control in Fig. 5E.<br><br>- Anti-dsRNA: Antibody was validated by manufacturer for IF and for detecting SARS-CoV-2 dsRNA in vero cells using mock and SARS-CoV-2 infected controls. Data was kindly provided by Autumn Grimins and John Connor, Boston University.<br>See https://www.cellsignal.com/products/primary-antibodies/dsrna-j2-mouse-mab/76651 |

## Eukaryotic cell lines

Policy information about cell lines and Sex and Gender in Research

| | |
|---|---|
| Cell line source(s) | BHK21 (ATCC), HEK293T (ATCC), Calu3 (ATCC), Vero cells stably overexpressing human TMPRSS2 (gifted from the Sean Whelan, WUSTL), Vero cells stably co-expressing human ACE2 and TMPRSS2 (gifted from A. Creanga and B. Graham at NIH). |
| Authentication | Cells were validated using controlled biological experiments for morphology, growth kinetics, and response to stimuli. |
| Mycoplasma contamination | All cell lines tested negative for Mycoplasma and are routinely tested every 3-6 months. |
| Commonly misidentified lines<br>(See ICLAC register) | None |

## Animals and other research organisms

Policy information about studies involving animals; ARRIVE guidelines recommended for reporting animal research, and Sex and Gender in Research

| | |
|---|---|
| Laboratory animals | C57BL/6 mice stably expressing hACE2 receptor (also known as K18 mice). Mice in the study were 6-8 weeks old. |
| Wild animals | Study did not involve any wild animals. |
| Reporting on sex | Information on sex was not collected. |
| Field-collected samples | Study did not involve samples collected from the field. |
| Ethics oversight | All protocols concerning animal use were approved (AN169239-01C) by the Institutional Animal Care and Use committees at the University of California, San Francisco and Gladstone Institutes and conducted in strict accordance with the National Institutes of Health Guide for the Care and Use of Laboratory Animal. |

Note that full information on the approval of the study protocol must also be provided in the manuscript.

# Dual use research of concern

Policy information about dual use research of concern

## Hazards

Could the accidental, deliberate or reckless misuse of agents or technologies generated in the work, or the application of information presented in the manuscript, pose a threat to:

| No | Yes | |
|---|---|---|
| ☒ | ☐ | Public health |
| ☒ | ☐ | National security |
| ☒ | ☐ | Crops and/or livestock |
| ☒ | ☐ | Ecosystems |
| ☒ | ☐ | Any other significant area |

## Experiments of concern

Does the work involve any of these experiments of concern:

| No | Yes | |
|---|---|---|
| ☒ | ☐ | Demonstrate how to render a vaccine ineffective |
| ☒ | ☐ | Confer resistance to therapeutically useful antibiotics or antiviral agents |
| ☒ | ☐ | Enhance the virulence of a pathogen or render a nonpathogen virulent |
| ☒ | ☐ | Increase transmissibility of a pathogen |
| ☒ | ☐ | Alter the host range of a pathogen |
| ☒ | ☐ | Enable evasion of diagnostic/detection modalities |
| ☒ | ☐ | Enable the weaponization of a biological agent or toxin |
| ☒ | ☐ | Any other potentially harmful combination of experiments and agents |
